# Genetic characteristics of waterfowl-origin H5N6 highly pathogenic avian influenza viruses and their pathogenesis in ducks and chickens

**DOI:** 10.3389/fmicb.2023.1211355

**Published:** 2023-06-15

**Authors:** Zhuoliang He, Xia Wang, Yu Lin, Siyu Feng, Xinyu Huang, Luxiang Zhao, Junsheng Zhang, Yangbao Ding, Weiqiang Li, Runyu Yuan, Peirong Jiao

**Affiliations:** ^1^College of Veterinary Medicine, Guangdong Laboratory for Lingnan Modern Agriculture, South China Agricultural University, Guangzhou, China; ^2^Guangdong Provincial Key Laboratory of Zoonosis Prevention and Control, Guangzhou, China; ^3^Guangdong Provincial Institution of Public Health, Guangdong Provincial Center for Disease Control and Prevention, Guangzhou, China

**Keywords:** H5N6 avian influenza virus, genetic evolution, pathogenicity, transmission, duck, chicken

## Abstract

Waterfowl, such as ducks, are natural hosts for avian influenza viruses (AIVs) and act as a bridge for transmitting the virus to humans or susceptible chickens. Since 2013, chickens and ducks have been threatened by waterfowl-origin H5N6 subtype AIVs in China. Therefore, it is necessary to investigate the genetic evolution, transmission, and pathogenicity of these viruses. In this study, we determined the genetic characteristics, transmission, and pathogenicity of waterfowl-origin H5N6 viruses in southern China. The hemagglutinin (*HA*) genes of H5N6 viruses were classified into the MIX-like branch of clade 2.3.4.4h. The neuraminidase (*NA*) genes belonged to the Eurasian lineage. The *PB1* genes were classified into MIX-like and VN 2014-like branches. The remaining five genes were clustered into the MIX-like branch. Therefore, these viruses belonged to different genotypes. The cleavage site of the HA proteins of these viruses was RERRRKR/G, a molecular characteristic of the H5 highly pathogenic AIV. The NA stalk of all H5N6 viruses contained 11 amino acid deletions at residues 58–68. All viruses contained 627E and 701D in the PB2 proteins, which were molecular characteristics of typical bird AIVs. Furthermore, this study showed that Q135 and S23 viruses could replicate systematically in chickens and ducks. They did not cause death in ducks but induced mild clinical signs in them. All the infected chickens showed severe clinical signs and died. These viruses were shed from the digestive and respiratory tracts and transmitted horizontally in chickens and ducks. Our results provide valuable information for preventing H5N6 avian influenza outbreaks.

## 1. Introduction

Avian influenza viruses (AIVs) can infect different types of animals, such as ducks, geese, chickens, quails, parrots, condors, pigs, horses, cats, dogs, tigers, seals, and humans (Buranathai et al., 2007; Jiao et al., 2012a,b,c; Long et al., 2019; Zhu et al., 2019; Zhuang et al., 2019; Puryear et al., 2023). As natural hosts for AIVs, ducks usually do not show clinical signs of AIV infection. Most AIV subtypes can be isolated from ducks, which facilitates the genetic recombination of the virus. Ducks often come into contact with terrestrial birds and even humans, which increases the chances of cross-host transmission of the AIVs (Cui et al., 2020).

In 1996, the first H5N1 subtype of highly pathogenic avian influenza virus (HPAIV) was discovered in domestic geese in Guangdong, south China (Xu et al., 1999). H5 HPAIVs have continued to circulate in birds in over 70 countries. The WHO/OIE/FAO classified the H5 AIV’s *HA* genes as Clades 0–9 (WHO, 2012). In 2006, clade 2 was subdivided into 2.1–2.5 (Neumann et al., 2010). In 2008, HA genes of clade 2 H5 virus were further divided into subclades 2.1.1–2.3.4. Since 2010, H5N1 HPAIV from clade 2.3.4 has recombined with many NA subtypes of viruses from waterfowl (including subtypes N2, N3, N5, N6, and N8) to produce several new viral subtypes. These viruses have been divided into four subclades, 2.3.4.1–2.3.4.4 (Bi et al., 2016; Du et al., 2017; Cui et al., 2020). The clade 2.3.4.4 was further separated into eight subclades, namely clades 2.3.4.4a–2.3.4.4h, owing to the evolution and spread of the virus worldwide (Gu et al., 2022). Clade 2.3.4.4a mainly refers to the H5N6 viruses that appeared in China in 2014. Clades 2.3.4.4b and 2.3.4.4c comprise H5N8 viruses that broke out in Eurasia and Africa between 2014 and 2018. Clade 2.3.4.4c includes the H5N8 AIVs that caused an avian flu outbreak in South Korea in 2014 and subsequently appeared in America and Europe and the H5N2 viruses that caused poultry outbreaks in the United States between 2014 and 2015. Clade 2.3.4.4e mainly refers to viruses that appeared in South Korea and Japan between 2016 and 2017. The viruses in Clade 2.3.4.4f and Clade 2.3.4.4 g were mainly H5N1 and H5N6 viruses found in China and the neighboring southwestern countries. H5N6 viruses of clades 2.3.4.4d and 2.3.4.4h were also isolated from samples in China (Cui et al., 2020; Li et al., 2020).

In April 2014, the first outbreak of H5N6 avian influenza in domestic poultry was recorded in Sichuan, China (Qu et al., 2019). Furthermore, H5N6 HPAIV has been widely concerned after a fatal human infection caused by a novel H5N6 virus in Sichuan, China, in 2014 (Pan et al., 2016). From 2014 to 2022, H5N6 AIVs caused 72 outbreaks in China, resulting in the death (or culling) of approximately 230,000 birds (Song et al., 2019; Siyu et al., 2023). The birds affected by the above outbreaks included chickens, ducks, geese, quails and wild birds such as wild geese, swans, mallards, northern shovelers, ferruginous pochards, great crested grebes, gadwalls and Eurasian wigeon (Cui et al., 2020). To prevent outbreaks of avian influenza outbreaks, we must continuously monitor the evolution of the H5N6 viruses and evaluate their pathogenicity and transmission.

Here, we isolated eight H5N6 AIVs from waterfowl during a routine surveillance of live poultry markets in southern China between 2017 and 2018. We analyzed the genetic characteristics of these viruses and selected the Q135 and S23 viruses to assess the pathogenicity and transmission of H5N6 AIVs in ducks and chickens.

## 2. Materials and methods

### 2.1. Viruses

As shown in Table 1, eight H5N6 HPAIVs (Q35, Q99, Q135, Q142, Q183, S23, SF227, and SF233) were isolated from waterfowl swabs from different live poultry markets of Guangdong Province, South China between 2017 and 2018. All swabs were collected in 1 mL of PBS, supplemented with penicillin and streptomycin. Subsequently, swab samples were shaken well and centrifuged at 10,000 g for 5 min. Then the supernatant was inoculated into 9-day-old SPF chicken embryo eggs that were cultured at 37°C for approximately 30 h to obtain viral allantoic fluid. The harvested allantoic fluid was centrifuged, dispensed into tubes, and stored at −80°C. Virus purification and 50% egg infective dose (EID_50_) determination were performed according to previous study (Thakur and Fezio, 1981; Jiao et al., 2018).

**Table 1:** H5N6 HPAIVs isolated between 2017 and 2018.

Virus	Abbreviation
A/duck/Guangdong/Q35/2017(H5N6)	Q35
A/goose/Guangdong/Q99/2017(H5N6)	Q99
A/goose/Guangdong/Q135/2017(H5N6)	Q135
A/duck/Guangdong/Q142/2017(H5N6)	Q142
A/goose/Guangdong/Q183/2017(H5N6)	Q183
A/duck/Guangdong/S23/2018(H5N6)	S23
A/goose/Guangdong/SF227/2018(H5N6)	SF227
A/goose/Guangdong/SF233/2018(H5N6)	SF233

### 2.2. Phylogenetic analysis

Viral RNA was extracted from the allantoic fluid using the RNA extraction kit (Vazyme, China) and reverse transcribed using reverse transcriptase (M-MLV; Vazyme). Eight AIV genes were amplified using universal primers (Wu et al., 2020). Purification of polymerase chain reaction products was accomplished using the DNA purification kit (ThermoFisher Scientific, United States). The polymerase chain reaction products were sequenced by Tsingke Biotechnology Co., Ltd. Sequencing data were compiled using the SeqMan program from Lasergene 7 (DNASTAR, Inc.). Phylogenetic trees of all viral genes were constructed using MEGA10.2 software (Sinauer Associates, Inc.), using the neighbor-joining method and the maximum complex likelihood model. The trees were assessed for reliability using 1,000 bootstrap replicates (Qu et al., 2019).

The accession numbers for the sequences of our H5N6 viruses are available from NCBI GenBank (OQ829384–OQ829391, OQ829392–OQ829399, OQ829408–OQ829415, OQ829425–OQ829432, OQ829632–OQ829639, OQ830450–OQ830457, OQ830486–OQ830493, and OQ830564–OQ830571).

### 2.3. Duck experiments

Seven-day-old, healthy, nonimmune Muscovy ducks were purchased from a farm in Guangdong, China, and raised in isolators. The ducks were serologically negative for avian influenza virus, monitored using the hemagglutination-inhibition (HI) test once a week for 3 weeks. Three weeks later, 18 ducks were divided equally into two groups and labeled individually, and each inoculation group (six ducks) was inoculated intranasally with 10^8^ EID_50_/0.1 mL of Q135 or S23 virus allantoic fluid. In addition, three ducks were inoculated with 0.1 mL of PBS as contact animals and were maintained with the inoculated ducks for 24 h after virus inoculation. On 3 DPI (day-post-inoculation), three ducks from the inoculated group were sacrificed, and the lungs, trachea, liver, spleen, kidneys, and brain were obtained. Each tissue sample (1 g) was cut and homogenized in 1 mL PBS containing antibiotics. The homogenate was centrifuged at 10,000 g for 5 min. After centrifugation, the virus titers of the supernatants were measured to check for viruses in the organs after inoculation into chicken embryos. The remaining ducks were observed for clinical signs up to 14 DPI. Additionally, oropharyngeal, and cloacal swabs were collected from all ducks at 3, 5, 7, 9, 11, and 13 DPI and suspended in 1 mL of PBS. The surviving ducks were euthanized after serum collection at 14 DPI. Seroconversion was confirmed using HI testing. Determination of HI antibodies in serum was performed as previously described (Jiao et al., 2018).

The supernatants of the tissue homogenates or swabs were 10-fold serially diluted, and 0.3 mL of each dilution was inoculated into three 9-day-old SPF chicken embryos (a rate of 0.1 mL/embryo). The embryos were cultured at incubator for 2 days. Positive allantoic fluid was determined using a hemagglutination test. Viral titers in the tissues and swabs were calculated according to a previous study(Thakur and Fezio, 1981; Jiao et al., 2018).

### 2.4. Chicken experiments

Four-week-old SPF chickens were purchased from Dahuanong Biotechnology Co., Ltd. in Guangdong, China, and raised in isolators. A total of 26 chickens were divided equally into two groups and labeled individually, and each inoculated group (eight chickens) was intranasally inoculated with 10^4^ EID_50_/0.1 mL of Q135 or S23 virus allantoic fluid. In addition, five chickens were inoculated with 0.1 mL as contact animals and were maintained with the inoculated chickens for 24 h after virus inoculation. At 2 DPI, three chickens of each inoculated group were euthanized, and the lungs, trachea, liver, spleen, kidneys, and brain were collected from three chickens of each inoculated group. Each tissue sample (1 g) was cut and homogenized in 1 mL of PBS containing antibiotics. The homogenate was centrifuged at 10,000 g for 5 min. After centrifugation, the virus titers of the supernatants were measured to check for viruses in the organs after inoculation into chicken embryos. The remaining chickens were observed for clinical signs up to 14 DPI. Alternatively, oropharyngeal, and cloacal swabs were collected from all chickens at 3, 5, 7, 9, 11, and 13 DPI and suspended in 1 mL of PBS. The methods of inoculating the supernatants of tissue homogenates or swabs and virus titer determination were based on an infection study in ducks.

### 2.5. Statistical analysis

Statistically differences in viral titers were determined using the Student’s *t*-test using the GraphPad 9.0. An analysis was considered statistically significant at the value of *p* < 0.05.

### 2.6. Ethics statement

All animal experiments were approved via the Laboratory Animal Management and Ethics Committee of South China Agricultural University and were conducted in the ABSL-3 facility (SCAUABSL2019-004).

## 3. Results

### 3.1. Phylogenic study of H5N6 AIVs

**Figure 1:**
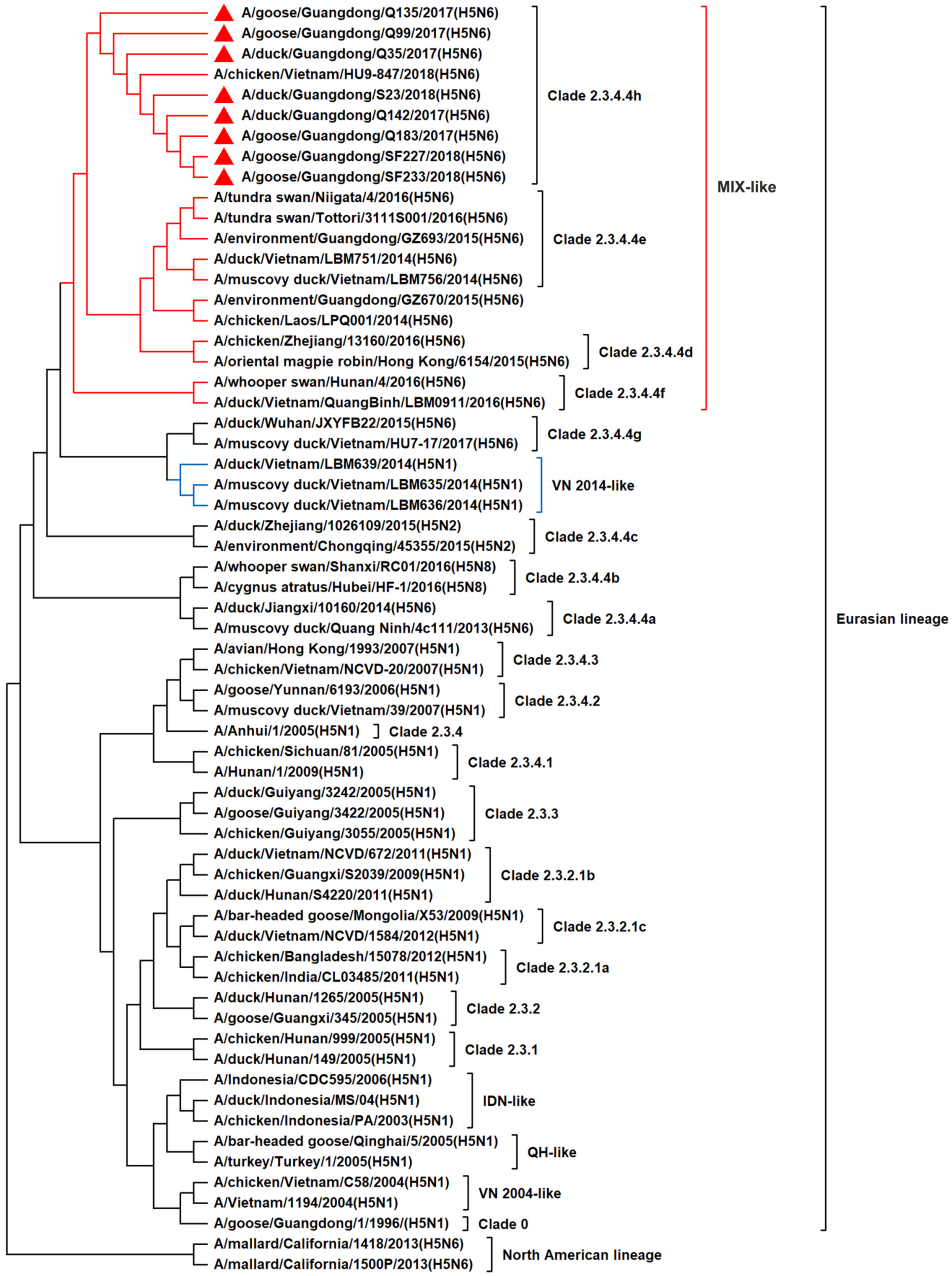
Phylogenetic relationships among H5 subtype AIVs based on their HA gene sequences (nucleotides 1–1,704). Our eight viruses have a triangle in front of the strain name. Qinghai, Guiyang, Vietnam, and Indonesia are abbreviated as QH, GY, VN, and IDN, respectively; MIX means these viruses come from China, Japan, Vietnam, and so on.

**Figure 2:**
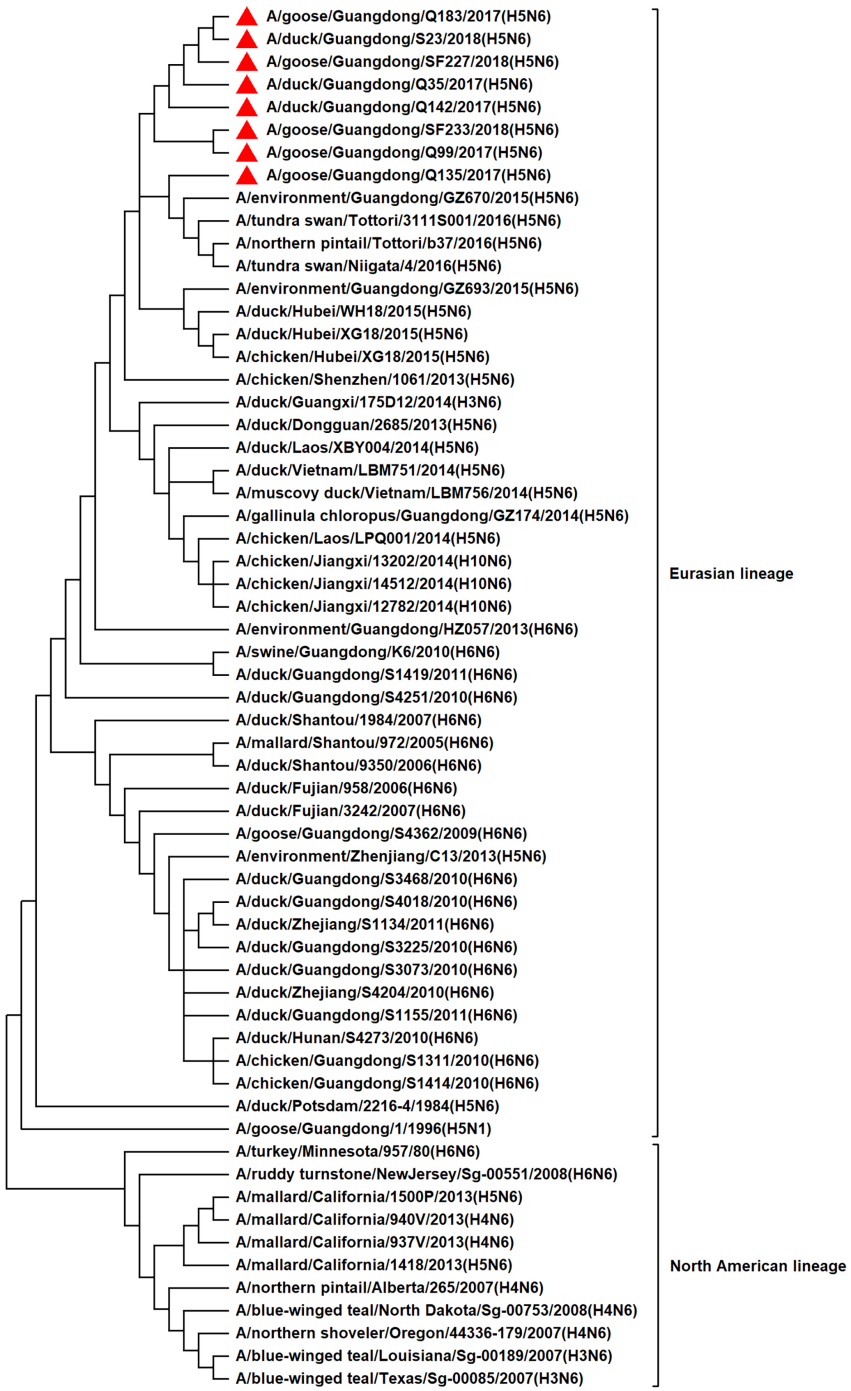
Phylogenetic relationships among N6 subtype AIVs based on their NA gene sequences (nucleotides 1–1,380). Our eight viruses have a triangle in front of the strain name.

**Figure 3:**
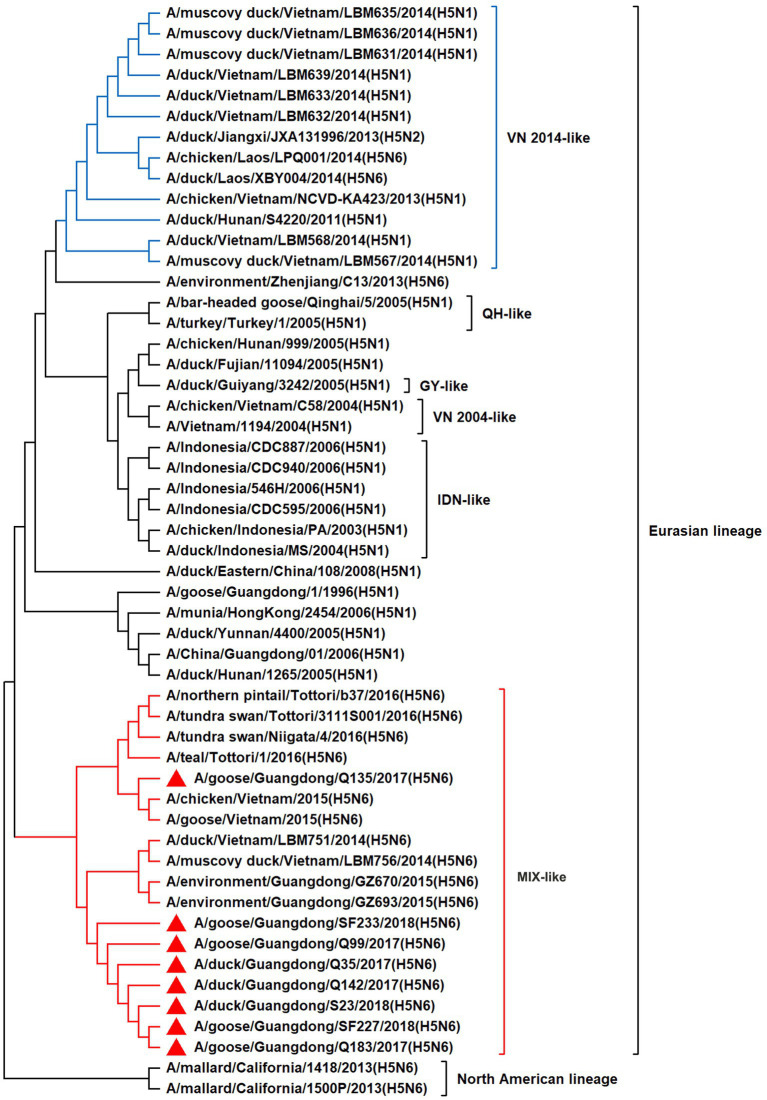
Phylogenetic relationships among H5 subtype AIVs based on their PB2 gene sequences (nucleotides 1–2,280). Our eight viruses have a triangle in front of the strain name. Qinghai, Guiyang, Vietnam, and Indonesia are abbreviated as QH, GY, VN, and IDN, respectively.

**Figure 4:**
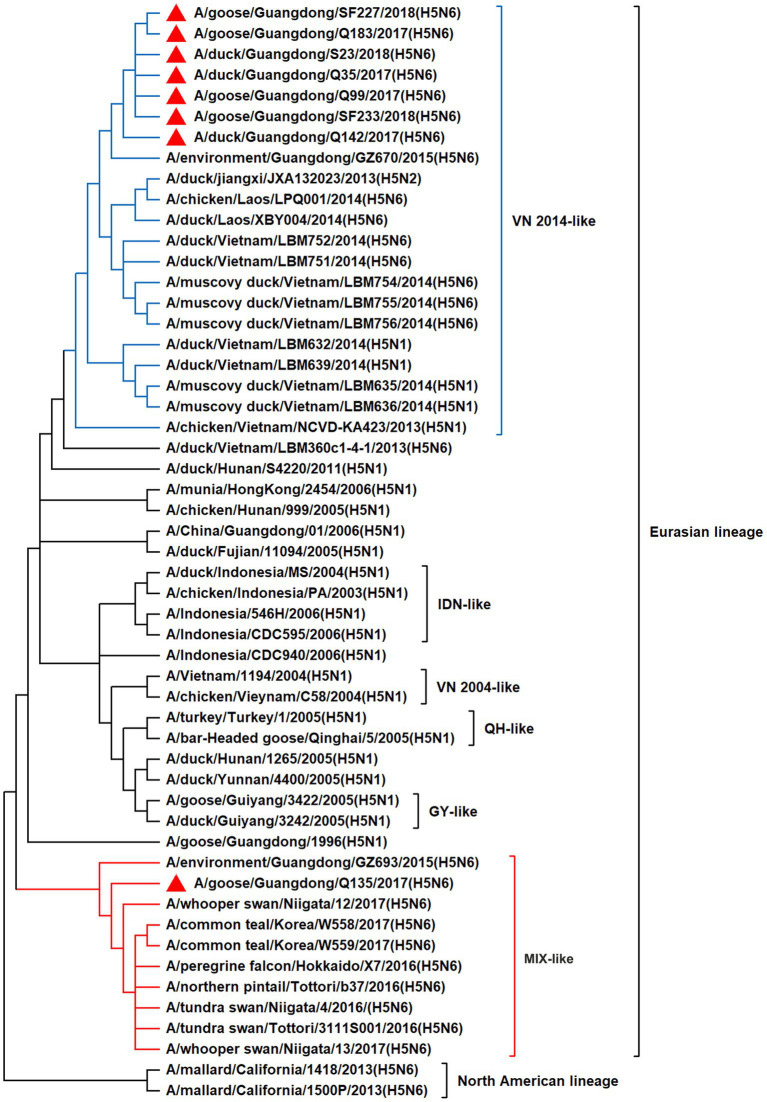
Phylogenetic relationships among H5 subtype AIVs based on their PB1 gene sequences (nucleotides 1–2,274). Our eight viruses have a triangle in front of the strain name. Qinghai, Guiyang, Vietnam, and Indonesia are abbreviated as QH, GY, VN, and IDN, respectively; MIX means these viruses come from China, Japan, Vietnam, and so on.

**Figure 5:**
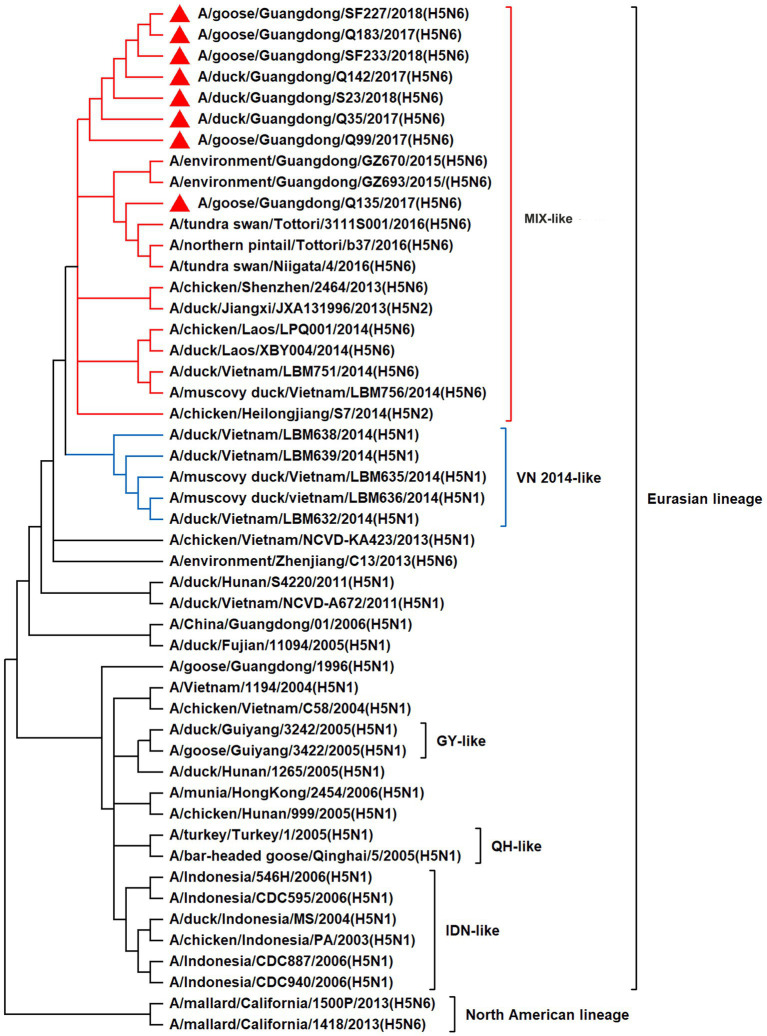
Phylogenetic relationships among H5 subtype AIVs based on their PA gene sequences (nucleotides 1–2,151). Our eight viruses have a triangle in front of the strain name. Qinghai, Guiyang, Vietnam, and Indonesia are abbreviated as QH, GY, VN, and IDN, respectively; MIX means these viruses come from China, Japan, Vietnam, and so on.

**Figure 6:**
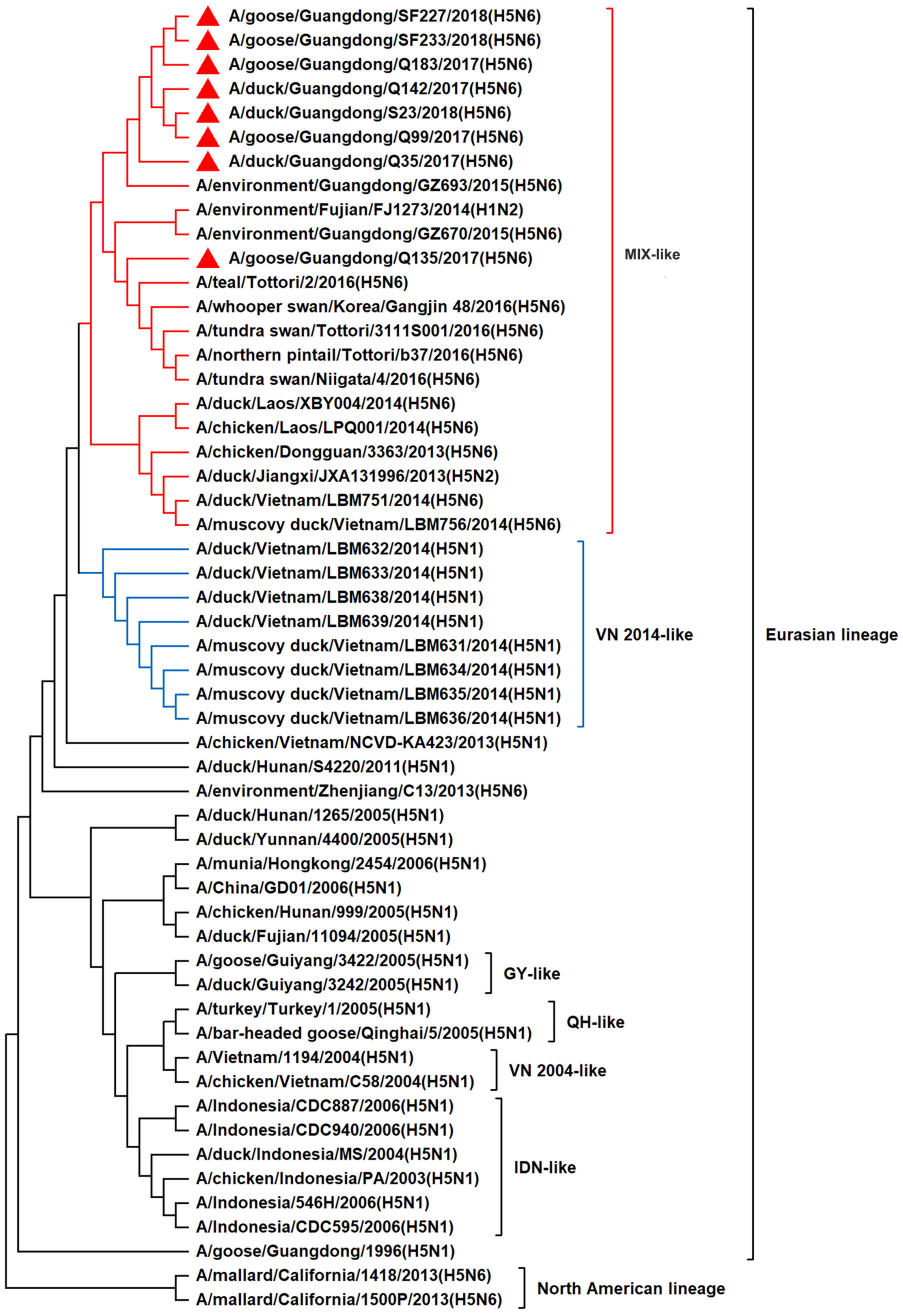
Phylogenetic relationships among H5 subtype AIVs based on their NP gene sequences (nucleotides 1–1,497). Our eight viruses have a triangle in front of the strain name. Qinghai, Guiyang, Vietnam, and Indonesia are abbreviated as QH, GY, VN, and IDN, respectively; MIX means these viruses come from China, Japan, Vietnam, and so on.

**Figure 7:**
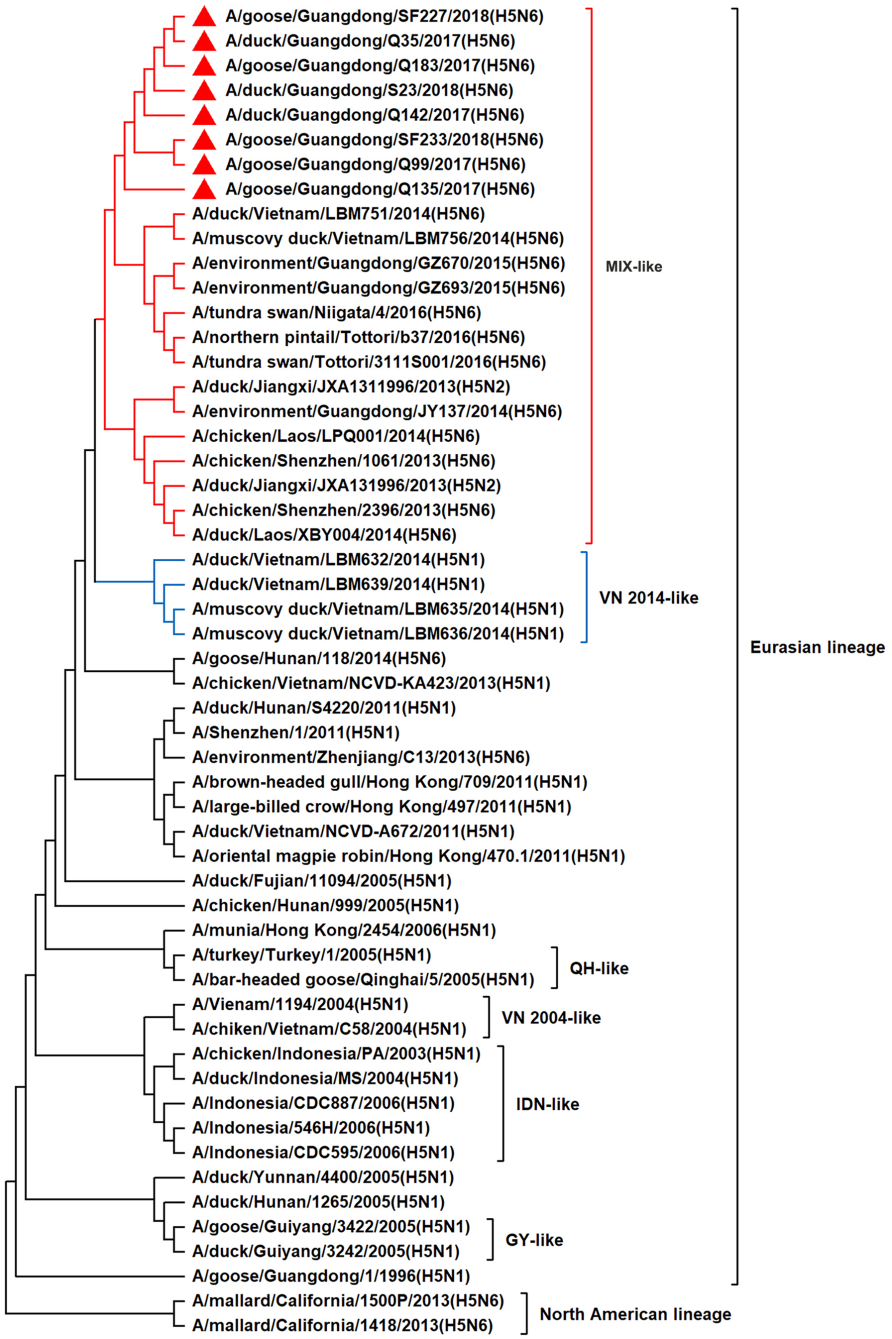
Phylogenetic relationships among H5 subtype AIVs based on their M gene sequences (nucleotides 1–982). Our eight viruses have a triangle in front of the strain name. Qinghai, Guiyang, Vietnam, and Indonesia are abbreviated as QH, GY, VN, and IDN, respectively; MIX means these viruses come from China, Japan, Vietnam and so on.

**Figure 8:**
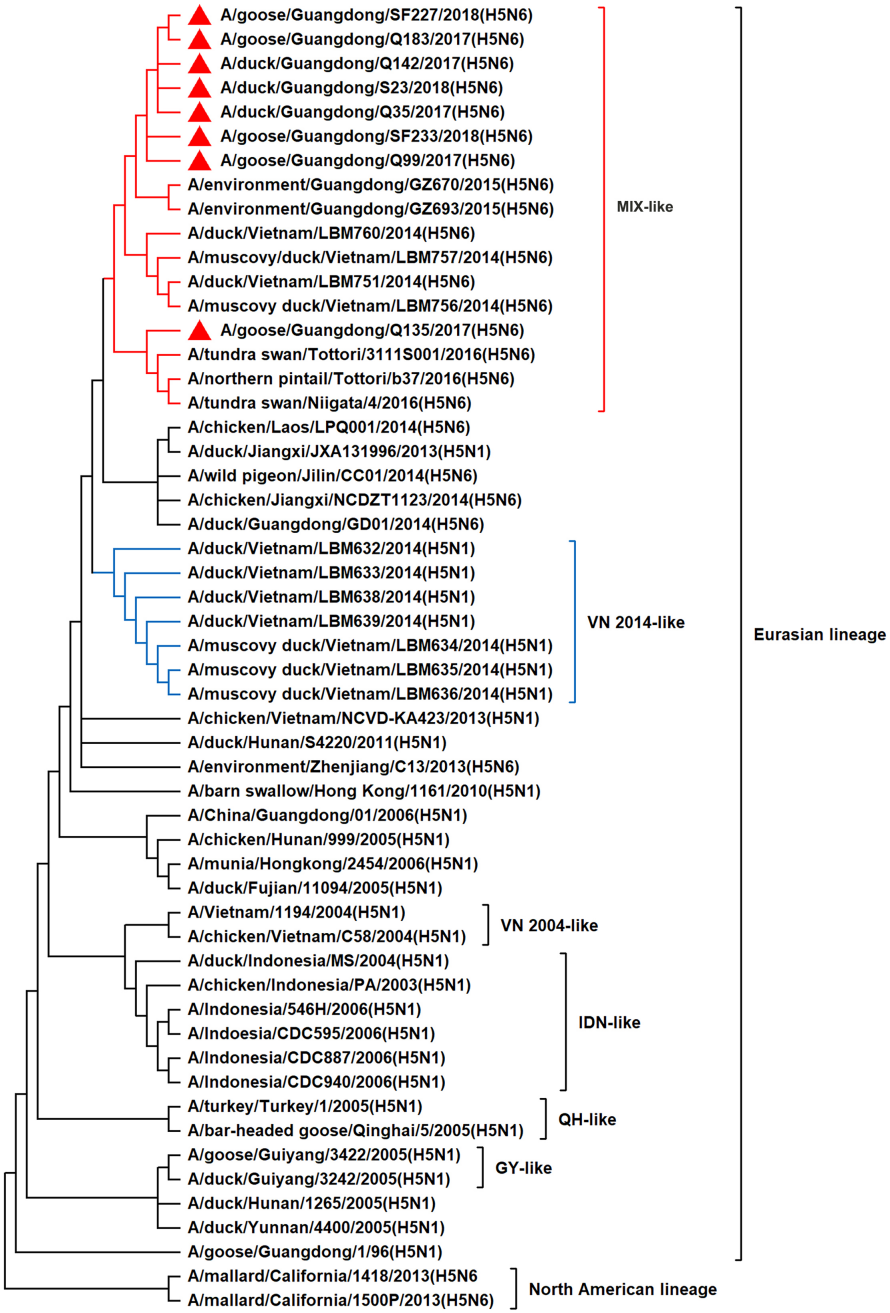
Phylogenetic relationships among H5 subtype AIVs based on their NS gene sequences (nucleotides 1–823). Our eight viruses have a triangle in front of the strain name. Qinghai, Guiyang, Vietnam, and Indonesia are abbreviated as QH, GY, VN, and IDN, respectively; MIX means these viruses come from China, Japan and Vietnam, and so on.

To resolve the genetic evolution of the eight H5N6 AIVs, viral genes were sequenced. We compared their sequences with those of representative viruses obtained from GenBank and performed a phylogenetic analysis. In this study, the *HA* genes of H5N6 AIVs were classified into MIX-like, Vietnam 2004-like (VN 2004-like), Vietnam 2014-like (VN 2014-like), Indonesia-like (IDN-like), Guiyang-like (GY-like), and Qinghai-like (QH-like). The *HA* genes of the H5N6 AIVs belonged to the MIX-like of Clade 2.3.4.4h (Figure 1). *NA* genes were divided into two branches: the Eurasian and North American lineages; the *NA* genes of our H5N6 AIVs were located in the Eurasian lineage (Figure 2). The *PB2* genes belonged to the MIX-like branch (Figure 3). The *PB1* genes were classified as MIX-like and VN 2014-like branches (Figure 4). The *PA*, *NP*, *M*, and *NS* genes were clustered into the MIX-like branch (Figures 5–8). In summary, the eight genes of the isolated AIVs belonged to different genotypes, indicating that their genetic evolution was diverse (Table 2).

**Table 2:** Summary of genetic reassortants of the H5N6 HPAIVs isolated between 2017 and 2018.

Virus	Gene							
	HA	NA	PB2	PB1	PA	NP	M	NS
Q35	MIX-like	Eurasian lineage	MIX-like	VN 2014-like	MIX-like	MIX-like	MIX-like	MIX-like
Q99	MIX-like	Eurasian lineage	MIX-like	VN 2014-like	MIX-like	MIX-like	MIX-like	MIX-like
Q135	MIX-like	Eurasian lineage	MIX-like	MIX-like	MIX-like	MIX-like	MIX-like	MIX-like
Q142	MIX-like	Eurasian lineage	MIX-like	VN 2014-like	MIX-like	MIX-like	MIX-like	MIX-like
Q183	MIX-like	Eurasian lineage	MIX-like	VN 2014-like	MIX-like	MIX-like	MIX-like	MIX-like
S23	MIX-like	Eurasian lineage	MIX-like	VN 2014-like	MIX-like	MIX-like	MIX-like	MIX-like
SF233	MIX-like	Eurasian lineage	MIX-like	VN 2014-like	MIX-like	MIX-like	MIX-like	MIX-like
SF227	MIX-like	Eurasian lineage	MIX-like	VN 2014-like	MIX-like	MIX-like	MIX-like	MIX-like

VN 2014-like stands for the Vietnam-like. MIX means these viruses come from China, Japan, Vietnam, and so on.

### 3.2. Molecular characteristics of H5N6 AIVs

To determine the molecular characteristics of the eight H5N6 viruses, their amino acid sequences were analyzed with reference to the genomic sequence of H5N1 AIV (A/goose/Guangdong/1/1996, GGD1-96). Herein, the cleavage sites in the HA protein of the eight H5N6 AIVs were RERRRKR/G, consistent with the characteristic of HPAIV. Compared with the reference strain GGD1-96, these viruses had amino acid deletions at positions 142 and 345. The amino acids related to the HA protein receptor-binding specificity were 190E, 225G, and 226-228QRG/QHG (the amino acid positions listed refer to H3 numbering). The HA protein of the GGD1-96 virus has seven potential glycosylation sites (26, 27, 39, 181, 302, 500, and 559). All seven potential glycosylation sites in our viruses were conserved; however, they added one potential glycosylation site at position 140 (NHT). NA proteins had five potential glycosylation sites (70, 86, 146, 201, and 402), but Q135 virus has an additional potential glycosylation site at 49 (NMS). The NA proteins had a deletion of 11 amino acids residues 58–68 in the stalk region of all isolated viruses. In the viral NA proteins, the amino acids were 119E, 152R, 275H, 293R, and 295 N, which correlated with the affinity of the active site and the neuraminidase inhibitor. The PB2 proteins in our viruses were the 627E and 701D. The PB1 proteins in our viruses were the 13P. The PA protein of the S23 virus was 383E, whereas that of the other seven viruses were 383D. A 479F amino acid residue of the NP proteins was observed in our viruses. The M1 proteins were found to have amino acid residues of 30D and 215A in all eight viruses. The amino acids at the amantadine/rimantadine resistance-associated sites of the M2 proteins were 26 L, 27 V, 30A, 31S, and 34G. Compared with the GGD1-96 NS1 proteins, our isolated viruses had these mutations (42S, 92E, 103F, 106 M, and 149A) and a five-amino-acid deletion at positions 83–87. In addition, their binding motif of the PDZ domain of the NS1 proteins was ESEV.

### 3.3. Pathogenicity and replication of the H5N6 HPAIVs isolated from waterfowl in ducks

To investigate the pathogenicity of H5N6 HPAIVs in ducks, we inoculated two groups of ducks with 10^8^ EID_50_/0.1 mL of Q135 and S23 viruses, respectively. At 3 DPI, three ducks from each inoculated group were euthanized to test whether the virus could cause a systemic infection. During the 14 DPI, all ducks were observed for clinical signs.

No ducks died after inoculation with the Q135 or S23 virus, but they presented mild clinical symptoms, such as low activity and loss of appetite, from 3 to 6 DPI. All human-infected ducks seroconverted at the 14 DPI, and HI antibody titers of the inoculated groups were 6–8 log_2_, which proved that all the inoculated ducks were infected with viruses. As shown in Table 3, we inoculated each grinding supernatant of the organs into chicken embryos and tested the viral replication titers of the Q135 in the lungs, trachea, liver, spleen, kidneys, and brain, which were 7.25, 6.58, 5.50, 4.75, 5.42, and 2.50 log_10_EID_50_/0.1 mL, respectively. The virus titers of S23 in the corresponding organs were determined using the same method and were 6.25, 6.33, 4.92, 3.75, 6.00, and 1.92 log_10_EID_50_/0.1 mL, respectively. Overall, the two viruses replicated in the respiratory, digestive, urinary, immune and nervous systems of the inoculated ducks. The virus titers of the Q135 virus were higher than those of S23 in the organs of the ducks, except in the kidneys.

**Table 3:** Replication of the H5N6 HPAIVs in ducks.

Virus	Virus titer (log_10_EID_50_/0.1 mL)	Virus titers in tissues on 3 DPI (log_10_EID_50_/0.1 mL) ± SD^a^ (positive numbers/total numbers)					
		Lung	Trachea	Liver	Spleen	Kidney	Brain
Q135	8.17	7.25 ± 0.66 (3/3)	6.58 ± 0.29 (3/3)	5.50 ± 0.00^*^ (3/3)	4.75 ± 0.66 (3/3)	5.42 ± 1.66 (3/3)	2.50 ± 1.73 (1/3)
S23	8.33	6.25 ± 0.66 (3/3)	6.33 ± 0.14 (3/3)	4.92 ± 0.29 (3/3)	3.75 ± 0.87 (3/3)	6.00 ± 0.66 (3/3)	1.92 ± 0.72 (1/3)

* Significant differences between virus titers in the corresponding tissues of ducks inoculated with the Q135 and S23 virus were determined by Student’s *t*-test. *p* < 0.05.

a A value of 1.5 was assigned if the virus was not detected from the undiluted supernatants in three chicken embryos (Li et al., 2005; Jiao et al., 2018). Virus titers were displayed as mean ± SD in log_10_EID_50_/0.1 mL of supernatants after centrifugation of grinding tissues.

These results indicated that Q135 and S23 viruses caused systemic infection and induced mild clinical signs, but do not cause death in inoculated and naturally infected ducks.

### 3.4. Transmission of the H5N6 HPAIVs isolated from waterfowl in ducks

To explore the virus shedding in infected ducks and the horizontal transmission of the H5N6 HPAIVs in the ducks, three ducks were intranasally inoculated with 0.1 mL of PBS, raised alongside the inoculated groups at 1 DPI, and designated as the contacted group. We obtained oropharyngeal and cloacal swabs from all ducks at 3, 5, 7, 9, 11, and 13 DPI. Then, we inoculated chicken embryos with the supernatants of the swabs from the inoculated and contacted ducks to determine their virus titers.

For the inoculated ducks, as shown in Table 4, Q135 virus titers ranged from 1.75–4.21 log_10_EID_50_/0.1 mL in the oropharyngeal swabs at the 3, 5, and 7 DPI, and 3.50–3.58 log_10_EID_50_/0.1 mL in the cloacal swab at the 3 and 5 DPI. S23 virus titers ranged from 3.17–4.46 log_10_EID_50_/0.1 mL in the oropharyngeal swabs at the 3 and 5 DPI, and 3.50–4.17 log_10_EID_50_/0.1 mL in the cloacal swabs at the 3 and 5 DPI. Q135-and S23-infected ducks shed the virus by 7 and 5 DPI, respectively. Despite the poor sustained shedding time of S23, monitoring data at 3 DPI showed that oropharyngeal and cloacal swabs had higher viral titers than those of Q135, suggesting that S23 was rapidly cleared from the ducks after infection. For the contacted ducks, two viruses were shed through the oropharynx until 9 DPI or 7 DPI (Q135 virus infection group was 1.58–4.33 log_10_EID_50_/0.1 mL and S23 virus infection group was 2.75–3.83 log_10_EID_50_/0.1 mL). The Q135 virus was tested using cloacal swabs at 3, 6, 9, 11, and 13 DPI (1.92–3.83 log_10_EID_50_/0.1 mL). Additionally, the S23 virus was tested using cloacal swabs at 3, 6, and 9 DPI (1.83–3.08 log_10_EID_50_/0.1 mL). Q135-infected ducks shed the virus by 13 DPI, with an infection peak infection at 7 DPI. By contrast, S23-infected ducks shed the undetectable virus on 11 DPI, with the peak of infection 2 days earlier than Q135. All contacted ducks survived and were seroconverted at the 14 DPI, and HI antibody titers of the contacted groups were 5–7 log2.

**Table 4:** Virus titers in swabs from inoculated and contacted ducks.

Virus	Infection group	3 DPI		5 DPI		7 DPI		9 DPI		11 DPI		13 DPI	
		Oro.	Clo.	Oro.	Clo.	Oro.	Clo.	Oro.	Clo.	Oro.	Clo.	Oro.	Clo.
Q135	Inoculated	4.21 ± 0.29 (6/6)	3.58 ± 0.72 (6/6)	3.5 ± 1.15 (3/3)	3.5 ± 1.52 (3/3)	1.75 ± 0.43 (1/3)	ND (0/3)	ND (0/3)	ND (0/3)	ND (0/3)	ND (0/3)	ND (0/3)	ND (0/3)
	Contacted	2.75 ± 0.50 (3/3)	ND (0/3)	4.25 ± 0.00 (3/3)	2.58 ± 1.27 (2/3)	4.33 ± 0.14 (3/3)	3.83 ± 1.15 (3/3)	1.58 ± 0.14 (1/3)	1.92 ± 0.52 (2/3)	ND (0/3)	2.08 ± 1.01 (1/3)	ND (0/3)	1.92 ± 0.72(1/3)
S23	Inoculated	4.46 ± 0.10 (6/6)	4.17 ± 0.82 (6/6)	3.17 ± 0.58 (3/3)	3.50 ± 0.0 (3/3)	ND (0/3)	ND (0/3)	ND (0/3)	ND (0/3)	ND (0/3)	ND (0/3)	ND (0/3)	ND (0/3)
	Contacted	2.83 ± 1.15 (2/3)	1.83 ± 0.58 (1/3)	3.83 ± 1.15 (3/3)	3.08 ± 1.51 (2/3)	2.75 ± 1.00 (3/3)	2.83 ± 1.53 (2/3)	ND (0/3)	1.83 ± 0.38 (2/3)	ND (0/3)	ND (0/3)	ND (0/3)	ND (0/3)

^*^Significant differences between virus titers in swabs of ducks infected with the Q135 and S23 virus were determined by Student’s *t*-test. *p* < 0.05. ^a^A value of 1.5 was assigned if the virus was not detected from the undiluted supernatants in three chicken embryos (Li et al., 2005; Jiao et al., 2018). Viral titers were displayed as mean ± SD in log_10_EID_50_/0.1 mL of swabs supernatants. Oro, oropharyngeal swabs. Clo, cloacal swabs. ND, not detected.

Therefore, both the Q135 and S23 viruses can be transmitted among ducks by direct contact. In particularly, the Q135 virus had a longer shedding time in contacted ducks than the S23 virus did.

### 3.5. Pathogenicity and replication of the H5N6 HPAIVs isolated from waterfowl in chickens

To understand the pathogenicity of the viruses in chickens, we inoculated two groups of chickens with 10^4^ EID_50_/0.1 mL of Q135 and S23 viruses. At 2 DPI, three chickens from each inoculated group were euthanized to test whether the virus could cause a systemic infection.

All chickens in the two groups displayed typical clinical symptoms before death, including decreased water and food intake, chicken claw edema, bleeding foot scales, oropharyngeal bleeding, greenish diarrhea, and even nervous signs. For Q135 virus, 3, 3, 1, and 1 inoculated chickens died at 2, 3, 4, and 5 DPI, respectively; 2 and 3 contacted chickens died at 3 and 4 DPI, respectively. For S23 virus, 5 and 3 inoculated chickens died at 2 and 3 DPI, respectively; 2, 2, and 1 contacted chickens died at 4, 5, and 6 DPI, respectively. As shown in Table 5, we inoculated each grinding supernatant of the organs into chicken embryos and tested the virus replication titers of Q135 in the lungs, trachea, liver, spleen, kidneys, and brain, and were 8.58, 8.00, 9.08, 8.17, 9.42, and 7.17 log_10_EID_50_/0.1 mL, respectively. Furthermore, the virus titers of S23 in the corresponding organs were determined using the same method, which were 9.00, 8.08, 8.42, 9.42, 9.75, and 8.58 log_10_EID_50_/0.1 mL, respectively. Overall, Q135 and S23 viruses replicated in the respiratory, digestive, urinary, immune, and nervous systems of the inoculated chickens. The viral titers of S23 were higher than those of Q135 in most chicken organs of chickens, except in the liver.

**Table 5:** Replication of the H5N6 HPAIVs in chickens.

Virus	Virus titer (log_10_EID_50_/0.1 mL)	Virus titers in tissues on 2 DPI (log_10_EID_50_/0.1 mL) ± SD^a^ (positive numbers/total numbers)					
		Lung	Trachea	Liver	Spleen	Kidney	Brain
Q135	8.17	8.58 ± 0.14 (3/3)	8.00 ± 0.66 (3/3)	9.08 ± 0.52 (3/3)	8.17 ± 2.31 (3/3)	9.42 ± 0.14 (3/3)	7.17 ± 0.63^*^ (3/3)
S23	8.33	9.00 ± 0.43 (3/3)	8.08 ± 0.29 (3/3)	8.42 ± 1.67 (3/3)	9.42 ± 0.14 (3/3)	9.75 ± 0.50 (3/3)	8.58 ± 0.14 (3/3)

* Significant differences between virus titers in the corresponding tissues of chickens inoculated with the Q135 and S23 virus were determined by Student’s *t*-test; *p* < 0.05.

a A value of 1.5 was assigned if the virus was not detected from the undiluted supernatants in three chicken embryos (Li et al., 2005; Jiao et al., 2018). Virus titers were displayed as mean ± SD in log_10_EID_50_/0.1 mL of supernatants after centrifugation of grinding tissues.

Hence, Q135 and S23 viruses caused severe systemic infections and 100% mortality in artificially inoculated and naturally infected chickens.

### 3.6. Transmission of the H5N6 HPAIVs isolated from waterfowl in chickens

The methods of assessing the viral shedding in infected chickens and the horizontal transmission of the H5N6 HPAIVs in the chickens were based on an infection study in ducks. As shown in Table 6, we found that virus titers were 3.80 log_10_EID_50_/0.1 mL and 4.15 log_10_EID_50_/0.1 mL in the oropharyngeal and cloacal swabs, respectively, from Q135-infected chickens at 3 DPI. Virus titers were 4.75 log_10_EID_50_/0.1 mL and 4.50 log_10_EID_50_/0.1 mL in the oropharyngeal and cloacal swabs, respectively, from Q135-infected chickens at 5 DPI. Virus titers were 4.75 log_10_EID_50_/0.1 mL and 4.50 log_10_EID_50_/0.1 mL in the oropharyngeal and cloacal swabs, respectively, from S23-infected chickens at 3 DPI. Additionally, the virus titers of oropharyngeal and cloacal swabs from contacted chicken were determined using the same method and were 4.75 log_10_EID_50_/0.1 mL and 4.50 log_10_EID_50_/0.1 mL, respectively, from Q135-infected chickens at 3 DPI. Virus titers were 2.10 log_10_EID_50_/0.1 mL, 2.45 log_10_EID_50_/0.1 mL in the oropharyngeal and cloacal swabs, respectively, from S23-infected chickens at 3 DPI. Virus titers were 4.42 log_10_EID_50_/0.1 mL and 4.25 log_10_EID_50_/0.1 mL in the oropharyngeal and cloacal swabs, respectively, from S23-infected chickens at 5 DPI.

**Table 6:** Virus titers in swabs from inoculated and contacted chickens.

Virus	Infection group	3 DPI		5 DPI		7 DPI		9 DPI		11 DPI		13 DPI	
		Oro.	Clo.	Oro.	Clo.	Oro.	Clo.	Oro.	Clo.	Oro.	Clo.	Oro.	Clo.
Q135	Inoculated	3.80 ± 1.30 (4/5)	4.15 ± 1.70 (4/5)	4.75 ± 0.00 (1/1)	4.50 ± 0.00 (1/1)	-	-	-	-	-	-	-	-
	Contacted	4.75 ± 0.40^*^ (5/5)	4.50 ± 0.25 (5/5)	-^b^	-	-	-	-	-	-	-	-	-
S23	Inoculated	4.75 ± 0.00 (3/3)	4.50 ± 0.25 (3/3)	-	-	-	-	-	-	-	-	-	-
	Contacted	2.10 ± 1.34 (1/5)	2.45 ± 1.37 (2/5)	4.42 ± 0.14 (3/3)	4.25 ± 0.43 (3/3)	-	-	-	-	-	-	-	-

* Significant differences between virus titers in the swabs of chickens infected with the Q135 and S23 virus were determined by Student’s *t*-test. *p* < 0.05.

^a^A value of 1.5 was assigned if the virus was not detected from the undiluted supernatants in three chicken embryos (Li et al., 2005; Jiao et al., 2018). Viral titers were displayed as mean ± SD in log_10_EID_50_/0.1 mL of swabs supernatants. ^b^All chickens died.

Thus, we demonstrated that Q135 and S23 were shed via the respiratory and digestive tracts and directly transmitted among chickens.

## 4. Discussion

H5 subtype viruses have accumulated over 27 genotypes through mutations in *HA* genes and have acquired *NA* genes from H3N2, H6N2, H4N6, H6N6, and H3N8 viruses and internal genes from H9N2, H7N9, and other unknown viruses (Zhuang et al., 2019; Cui et al., 2020). Since 2013, emerging H5N6 HPAIVs have spread from Asia to other countries via western and eastern routes, causing diseases in birds and humans (Bi et al., 2016). From 2014 to 2022, Clade 2.3.4.4a–h H5N6 HPAIVs caused the death (or culling) of approximately 230,000 birds in China (Song et al., 2019; Cui et al., 2020; Siyu et al., 2023). In addition, there were 65 cases of human infection with the H5N6 virus in China from 2014 to 2021, 64 of which occurred in southern China. These viruses belonged to clade 2.3.4.4a, 2.3.4.4b, 2.3.4.4d, 2.3.4.4g, and 2.3.4.4h (Zhu et al., 2022). In this study, eight H5N6 HPAIVs were isolated from domestic waterfowl from 2017 to 2018, in southern China. The *HA* genes of these viruses belonged to the MIX-like branch of clade 2.3.4.4h, whereas the *NA* genes belonged to the Eurasian lineage. The *PB2* genes were also categorized into the MIX-like branch, and the *PB1* genes were classified as MIX-like and VN 2014-like branches. The *PA*, *NP*, *M*, and *NS* genes of the H5N6 viruses were clustered into a MIX-like branch (Figures 1–8). This suggests that Clade 2.3.4.4h H5N6 HPAIV in south China underwent genetic recombination.

Since their initial emergence, H5N6 HPAIVs have undergone further genetic evolution owing to the accumulation of mutations. These mutations could lead to changes in the receptor-binding properties, virulence, and antigenicity of the virus, thereby affecting its ability to infect and spread between hosts. In the present study, the isolated strains had RERRRKR/G sequences at the HA cleavage sites and they were 100% lethal to chickens, which indicated they were HPAIVs (Huang et al., 2021). In our strains, the amino acid residues related to HA protein receptor-binding site were 190E, 225G, and 226–228QRG/QHG, indicating that the viruses tended to bind to the α-2-3-galactoside sialic acid receptor. All our viruses were mutated to a potential glycosylation site at position 140 (NHT) of HA proteins and the Q135 virus added one potential glycosylation site at position 49 (NMS) of the NA protein, which may lead to immune escape by interfering with antibody recognition(Wagner et al., 2000; Zhao et al., 2017). The deletion of 11 amino acids from residues 58–68 in the stalk region of NA proteins has been associated with increased viral virulence in mammals (Matsuoka et al., 2009; Fan et al., 2014). Fortunately, their NA and M2 proteins did not occur resistant mutation of neuraminidase inhibitors or Amantadine (Fan et al., 2014; Moorthy et al., 2014). The 627 K and 701 N mutations in PB2 enhance viral replication and pathogenicity in mammals(Hatta et al., 2001; Li et al., 2005). However, all our viruses were E at the 627 site and D at the 701 site of the PB2 proteins, which are molecular characteristics of typical AIVs from birds. The L13P of the PB1 proteins, D383K of PA, L479F substitution in the NP, and 30D and 215A of M1 were present in all our viruses, and these mutations helped to enhance their viral pathogenicity in mammals (Gabriel et al., 2008; Long et al., 2008; Fan et al., 2009; Song et al., 2011, 2015; Yang et al., 2021). The NS1 proteins have these mutations P42S, D92E, L103F, I106M, and V149A and a five-amino-acid deletion at positions 83–87 compared with GGD1-96 (Long et al., 2008), which are the molecular characterization that enhance viral virulence (Donelan et al., 2003; Seo et al., 2004; Banet-Noach et al., 2007; Jiao et al., 2008). In addition, their binding motif of the PDZ domain of the NS1 proteins is ESEV, indicating that they were avian-origin AIVs (Obenauer et al., 2006; Jackson et al., 2008). Thus, the H5N6 viruses in this study have some molecular characteristics contributing to enhanced pathogenicity in avians or mammals.

Ducks are natural hosts of AIVs, and almost all AIV subtypes can be isolated from ducks (Chen et al., 2006; Deng et al., 2013). However, no AIVs, including the highly pathogenic H5 strain, were known to cause disease symptoms or death in ducks before 2002 (Webster et al., 1992; Chen et al., 2004). In the late 20th century, researchers discovered that AIVs do not cause disease in ducks but mainly infect monolayer columnar epithelial cells in their colon, using virus titers as high as 10^7–8^ EID_50_/mL (Webster et al., 1978; Kida et al., 1980). Additionally, early H5N1 AIVs could not replicate systemically or induce strong immune responses in ducks (Burggraaf et al., 2014; Kuchipudi et al., 2014). However, the H5N1 virus, isolated from wild waterfowl in 2002, caused severe neurological symptoms in ducks, ultimately killing them (Sturm-Ramirez et al., 2004). From April to June 2005, there was a large outbreak of H5N1 infection among migratory waterfowl in Qinghai Lake, western China (Chen et al., 2005; Liu et al., 2005). The H5N1 viruses isolated in 2004, 2008, and 2012, and the H5N6 virus isolated between 2014 and 2016 could replicate in multiple organs of ducks, such as lungs, trachea, liver, spleen, kidneys, pancreas, intestines, and brain and even cause death (Wei et al., 2013; Yuan et al., 2014; Uchida et al., 2019;Wu et al., 2019; Huang et al., 2021). Moreover, Wei found that H5N1 highly pathogenic viruses induce an excessive immune response resulting in immune damage, multiple system organ failure, and disease exacerbation in ducks (Wei et al., 2013). In our study, we selected Q135 and S23 viruses for the pathogenicity study based on the sequencing results of eight viral genes, differences in time and host of the virus isolated. The two H5N6 HPAIVs (Q135 and S23) replicated in the lungs, trachea, liver, pancreas, intestine, spleen, cloacal bursa, trachea, kidneys, and brain, but were not lethal to ducks; it exhibited mild clinical signs for 3–6 DPI. Overall, the two viruses replicated in the respiratory, digestive, urinary, immune, and nervous systems of the inoculated ducks. Additionally, there were differences in the horizontal transmission patterns of viruses isolated at different stages in the duck populations. In the 1980s, ducks infected with AIV shed the virus only through the cloaca. The virus did not spread widely among the duck population (Webster et al., 1978; Kida et al., 1980). In 2002, H5N1 viruses were isolated from the drinking water of diseased and contacted ducks, demonstrating the effective transmission of the virus (Sturm-Ramirez et al., 2004). Recent studies have shown that infected ducks shed H5N6 AIVs through their digestive and respiratory tracts (Wu et al., 2019; Huang et al., 2021). In this study, inoculated and contacted ducks shed the H5N6 virus through the oropharynx and cloaca. These two viruses are also horizontally transmitted in ducks through contact. Ducks have a long shedding time when infected with H5N6 HPAIVs; this is concerning, since they could be a potentially effective source of avian influenza transmission.

Almost all waterfowl-origin AIVs are weakly pathogenic to ducks. However, these waterfowl-origin viruses are highly pathogenic when transmitted from waterfowl to chickens. In chickens, these viruses cause severe inflammation and tissue damage to most organs, including the respiratory and digestive systems. The clinical signs of infected chickens include respiratory distress, lethargy, reduced egg production, and sudden death. The severity of avian influenza in chickens depends on various factors, including age, breed, immune status, and the virus strain (Webster et al., 1992; Yoon et al., 2014). Most strains of the H5N6 virus are highly pathogenic, causing a mortality rate of up to 100% in infected flocks. Since 1996, there has been a trend toward increased pathogenicity of H5 HPAIV in chickens. A low dose (10^3^ EID_50_/0.1 mL) of the H5N1 virus GGD1-96 strain did not cause death in inoculated chickens; however, the same dose of H5N1 HPAIV isolated in 2012 caused illness and death in inoculated chickens (Yuan et al., 2014). The H5 subtype HPAIV induces the overexpression of cytokines such as IFN-I, IL-6, OAS, IL-8, and Mx1 in infected chickens, causing immune damage and an excessive inflammatory response, which accelerates the death of infected chickens (Wei et al., 2013; Song et al., 2019; Chen et al., 2020). In this study, two H5N6 HPAIVs (Q135 and S23) replicated in all tested tissues of chickens. Overall, both viruses replicated in the respiratory, digestive, urinary, immune, and nervous systems of the inoculated chickens. Infected chickens exhibited typical avian influenza symptoms from 2 DPI, and all inoculated and contacted chickens died within 3–5 DPI. In addition to strain differences, the horizontal transmission of H5 subtype viruses among contacted chickens is related to the dose of the infected virus in the inoculated chickens. When chickens were inoculated with H5N1 HPAIV at 10^3^ EID_50_/0.1 mL, they showed mild clinical signs and undetectable viral shedding. When chickens were inoculated with H5N1 or H5N6 HPAIV at 10^6^ EID_50_/0.1 mL, those in the direct contact group began shedding the virus at 2 DPI and eventually died (Yuan et al., 2014; Jiao et al., 2016). However, some H5N6 AIVs do not kill chickens but spread silently through flocks (Mei et al., 2019; Uchida et al., 2019). The H5N6 HPAIVs that have become prevalent in recent years are mainly transmitted in bird populations through direct contact with droppings or through the oral and nasal secretions of infected birds (Qu et al., 2019; Chen et al., 2020). The H5N6 virus can also be transmitted via air and aerosols in crowded flocks, such as in crowded live-bird markets (Zhou et al., 2016). In this study, inoculated and contacted chickens shed viruses via the oropharynx and cloaca, confirming that both H5N6 HPAIVs were transmitted horizontally in chickens.

In summary, waterfowl-origin clade 2.3.4.4h H5N6 HPAIVs displayed genetic diversity in their phylogenetic analysis. Our H5N6 viruses replicated systematically, causing severe and mild clinical symptoms in chickens and ducks, respectively. These viruses are shed from the infected poultry and transmitted horizontally in chickens and ducks. These findings provide a basis for preventing H5N6 avian influenza outbreaks.

## Data availability statement

The data presented in the study are deposited in the NCBI GenBank repository, accession numbers OQ829384–OQ829391, OQ829392–OQ829399, OQ829408–OQ829415, OQ829425–OQ829432, OQ829632–OQ829639, OQ830450–OQ830457, OQ830486–OQ830493 and OQ830564–OQ830571.

## Ethics statement

The animal study was reviewed and approved by the Laboratory Animal Management and Ethics Committee of South China Agricultural University.

## Author contributions

ZH, XW, and PJ designed and performed the main experiments. YL, SF, XH, LZ, JZ, YD, and WL performed the experiments. ZH, PJ, and RY drafted the manuscript. All authors contributed to the article and approved the submitted version.

## Funding

This work was supported by grants from the National Key Research and Development Program of China (2021YFD1800202), the National Natural Science Foundation of China (32072844), and the Laboratory of Lingnan Modern Agriculture Project (NT2021007).

## Conflict of interest

The authors declare that the research was conducted in the absence of any commercial or financial relationships that could be construed as a potential conflict of interest.

## Publisher’s note

All claims expressed in this article are solely those of the authors and do not necessarily represent those of their affiliated organizations, or those of the publisher, the editors and the reviewers. Any product that may be evaluated in this article, or claim that may be made by its manufacturer, is not guaranteed or endorsed by the publisher.
